# Supplementary material for: Structural differences between REM and non-REM dream reports assessed by graph analysis
Source: PLoS One. 2020 Jul 23;15(7):e0228903. doi: 10.1371/journal.pone.0228903 (PMC7377375; doi:10.1371/journal.pone.0228903)
Supplement: S1 Text — (DOCX) [file pone.0228903.s001.docx]

**S1 Text. Overview of Levels of Perceptual Interaction Rating Scale (PIRS)**

- (0) “No Recall”. A score of 0 refers to a situation in which the participant reports having experienced no mental experiences.
- (1) “White Dream”. A score of 1 is given to a situation in which the participant feels as if they were dreaming but cannot remember any details of the dream.
- (2-3) “Nonvisual Recall (Conceptual)”. A rating of 2-3 refers to non-visual experiences that typically involve conceptual or thought-like mentation. A score of 2 is given to simple, fragmented experiences that are isolated and lack coherence, while a score of 3 is given to more complex, ongoing coherent experiences, which may be accompanied by non-visual perceptions such as sounds (voices or music), smells or kinesthetic sensations.
- (4-5) “Visual/Perceptual, Non-Narrative/Incoherent”. A rating of 4-5 includes experiences with visual perception that take place within an isolated scene/incident that is unrelated to an ongoing narrative. A rating of 4 is given to the most basic of visual experiences that do not involve any interactions, while a rating of 5 is scored for visual experience with an isolated interaction with other characters or with the dream environment.
- (6-9) “Visual/Perceptual Dreaming, Part of an Ongoing Narrative”. A rating of 6-9 refers to experiences that take place within a visual/perceptual dream environment and describe one or more interactions that are related to an ongoing series of events or a story/narrative. The numbers increase in accordance with the complexity of the narrative described in the report, relating to the number of stages of development (6 - one, 7 - one or two, 8 - three or four, 9 - five or more) and the intensity of the interactions present (6 - limited interaction, 7 - moderate interaction, 8 - prolonged interaction, 9 - intensive interaction).
