# Supplementary material for: Structural differences between REM and non-REM dream reports assessed by graph analysis
Source: PLoS One. 2020 Jul 23;15(7):e0228903. doi: 10.1371/journal.pone.0228903 (PMC7377375; doi:10.1371/journal.pone.0228903)
Supplement: S1 Table — (DOCX) [file pone.0228903.s002.docx]

**S2 Table. Correlation Matrix Showing Relationship Between Variables of Interest.**

|  |  | Connectedness  (Original Graph) | | | Connectedness  (Sliding Window) | | | Random-likeness | | Total Recall Count | Report Features | |
| --- | --- | --- | --- | --- | --- | --- | --- | --- | --- | --- | --- | --- |
|  |  | Edges | LCC | LSC | Edges | LCC | LSC | LCCz | LSCz | TRC | Word Count | No. Paragraphs |
| Connectedness (Original Graph) | Edges | ----- |  |  |  |  |  |  |  |  |  |  |
|  | LCC | .956 | ----- |  |  |  |  |  |  |  |  |  |
|  | LSC | .948 | .975 | ----- |  |  |  |  |  |  |  |  |
| Connectedness (Sliding Window) | Edges | .181 | .183 | .301 | ----- |  |  |  |  |  |  |  |
|  | LCC | -.009 | .134 | .197 | .469 | ----- |  |  |  |  |  |  |
|  | LSC | .075 | .053 | .161 | .776 | .335 | ----- |  |  |  |  |  |
| Randomlikeness | LCCz | -.328 | -.142 | -.152 | .048 | .480 | -.054 | ----- |  |  |  |  |
|  | LSCz | -.324 | -.156 | -.091 | .285 | .579 | .229 | .571 | ----- |  |  |  |
| Total Recall Count | TRC | .681 | .729 | .729 | .389 | .257 | .224 | -.048 | .012 | ------ |  |  |
| Report Features | Word Count | .998 | .958 | .937 | .138 | -.032 | .040 | -.341 | -.337 | .670 | ----- |  |
|  | No. Paragraphs | .283 | .243 | .117 | -.780 | -.436 | -.518 | -.291 | -.442 | -.080 | .327 | ----- |

Note* values are given as Spearman’s (rho) coefficient. Values that reach statistical significance (α < .05) are shown in red.
