# Supplementary material for: Structural differences between REM and non-REM dream reports assessed by graph analysis
Source: PLoS One. 2020 Jul 23;15(7):e0228903. doi: 10.1371/journal.pone.0228903 (PMC7377375; doi:10.1371/journal.pone.0228903)
Supplement: S1 Appendix — (DOCX) [file pone.0228903.s003.docx]

## **S3 Appendix. Showing Results for Follow-up Analysis Controlling for the Occurrence of Common Words (via NLTK List).**

## **S3A Table**. **Output from Wilcoxon Sign-Rank Test.**

|  | REM | N2 | Z-score | effect size (r) | p |
| --- | --- | --- | --- | --- | --- |
| Edges | 28.2 ± 1.11 | 27.91 ± 1.23 | -2.45 | .398 | .014 |
| LCC | 22.03 ± 1.51 | 20.33 ± 2.26 | -2.45 | .398 | .014 |
| LSC | 16.15 ± 3.66 | 14.96 ± 3.09 | -1.84 | .298 | .066 |

*Note:* Values that reach statistical significance (α < .05) are shown in red.

**S3B Table. Output from Generalised Linear Mixed Model Predicting Sleep Stage.**

| Individual Predictors | Pseudo R^2^ | Pseudo R^2^ Change | p |
| --- | --- | --- | --- |
| **LCC** | .081 | .081 | .011 |
| Composite Models | Pseudo R^2^ | Pseudo R^2^ Change | p |
| LCC + **TRC** | .144 | .069 | .020 |
| TRC + **LCC** | .144 | .037 | .094 |

*Note:* Values that reach statistical significance (α < .05) are shown in red. Pseudo R^2^ Change

and significance test reflects the contribution of the predictor highlighted in bold.

**S3C Table. Output for Cumulative Link Mixed Models Predicting PIRS Ratings.**

| Individual Predictors | Pseudo R^2^ | Pseudo R^2^ Change | p |
| --- | --- | --- | --- |
| Sleep Stage + **Edges** | .203 | .086 | .002 |
| Sleep Stage + **LCC** | .361 | .267 | <.001 |
| Sleep Stage + **LSC** | .211 | .096 | .001 |
| Sleep Stage + **LSCz** | .350 | .255 | <.001 |
| Composite Models: | Pseudo R^2^ | Pseudo R^2^ Change | p |
| Sleep Stage + TRC + **LCC** | .646 | .214 | .001 |
| Sleep Stage + TRC + **LSCz** | .646 | .162 | <.001 |

*Note:* Values that reach statistical significance (α < .05) are shown in red. Pseudo R^2^ Change

and significance test reflects the contribution of the predictor highlighted in bold.
