# Supplementary material for: Structural differences between REM and non-REM dream reports assessed by graph analysis
Source: PLoS One. 2020 Jul 23;15(7):e0228903. doi: 10.1371/journal.pone.0228903 (PMC7377375; doi:10.1371/journal.pone.0228903)
Supplement: S2 Appendix — (DOCX) [file pone.0228903.s004.docx]

**S4 Appendix. Showing Results from Follow-up Analysis Partialling Out Number of Paragraphs.**

**S4A Table. Output from Generalised Linear Mixed Model Predicting Sleep Stage.**

| Individual Predictors | Pseudo R^2^ | Pseudo R^2^ Change | p |
| --- | --- | --- | --- |
| Paragraphs + **LCC** | .058 | .058 | .017 |
| Composite Models | Pseudo R^2^ | Pseudo R^2^ Change | p |
| Paragraphs + TRC+ **LCC** | .138 | .037 | .062 |
| Paragraphs + LCC + **TRC** | .138 | .074 | .007 |

*Note:* Values that reach statistical significance (α < .05) are shown in red. Pseudo R^2^ Change

and significance test reflects the contribution of the predictor highlighted in bold.

**S4B Table. Output from Cumulative Link Mixed Models Predicting PIRS Ratings.**

| Individual Predictors | Pseudo R^2^ | Pseudo R^2^ Change | p |
| --- | --- | --- | --- |
| Paragraphs + Sleep Stage + **Edges** | .194 | .047 | .013 |
| Paragraphs + Sleep Stage + **LCC** | .229 | .088 | <.001 |
| Paragraphs + Sleep Stage + **LSC** | .180 | .031 | .046 |
| Paragraphs + Sleep Stage + **LSCz** | .177 | .027 | .061 |
| Composite Models: | Pseudo R^2^ | Pseudo R^2^ Change | p |
| Paragraphs + Sleep Stage + TRC + **LCC** | .646 | .055 | .008 |
| Paragraphs + Sleep Stage + TRC + **LSCz** | .646 | .060 | .006 |

*Note:* Values that reach statistical significance (α < .05) are shown in red. Pseudo R^2^ Change

and significance test reflects the contribution of the last predictor.
